# Utilizing a Personal Smartphone Custom App to Assess the Patient Health Questionnaire-9 (PHQ-9) Depressive Symptoms in Patients With Major Depressive Disorder

**DOI:** 10.2196/mental.3889

**Published:** 2015-03-24

**Authors:** John Torous, Patrick Staples, Meghan Shanahan, Charlie Lin, Pamela Peck, Matcheri Keshavan, Jukka-Pekka Onnela

**Affiliations:** ^1^ Harvard Longwood Psychiatry Residency Training Prorgam Boston, MA United States; ^2^ Beth Israel Deaconess Medical Center Department of Psychiatry Harvard Medical School Boston, MA United States; ^3^ Department of Biostatistics Harvard School of Public Health Harvard University Boston, MA United States; ^4^ Pocket Gems Palo Alto, CA United States

**Keywords:** medical informatics, mobile health, depression

## Abstract

**Background:**

Accurate reporting of patient symptoms is critical for diagnosis and therapeutic monitoring in psychiatry. Smartphones offer an accessible, low-cost means to collect patient symptoms in real time and aid in care.

**Objective:**

To investigate adherence among psychiatric outpatients diagnosed with major depressive disorder in utilizing their personal smartphones to run a custom app to monitor Patient Health Questionnaire-9 (PHQ-9) depression symptoms, as well as to examine the correlation of these scores to traditionally administered (paper-and-pencil) PHQ-9 scores.

**Methods:**

A total of 13 patients with major depressive disorder, referred by their clinicians, received standard outpatient treatment and, in addition, utilized their personal smartphones to run the study app to monitor their symptoms. Subjects downloaded and used the Mindful Moods app on their personal smartphone to complete up to three survey sessions per day, during which a randomized subset of PHQ-9 symptoms of major depressive disorder were assessed on a Likert scale. The study lasted 29 or 30 days without additional follow-up. Outcome measures included adherence, measured by the percentage of completed survey sessions, and estimates of daily PHQ-9 scores collected from the smartphone app, as well as from the traditionally administered PHQ-9.

**Results:**

Overall adherence was 77.78% (903/1161) and varied with time of day. PHQ-9 estimates collected from the app strongly correlated (*r*=.84) with traditionally administered PHQ-9 scores, but app-collected scores were 3.02 (SD 2.25) points higher on average. More subjects reported suicidal ideation using the app than they did on the traditionally administered PHQ-9.

**Conclusions:**

Patients with major depressive disorder are able to utilize an app on their personal smartphones to self-assess their symptoms of major depressive disorder with high levels of adherence. These app-collected results correlate with the traditionally administered PHQ-9. Scores recorded from the app may potentially be more sensitive and better able to capture suicidality than the traditional PHQ-9.

## Introduction

Depression is a highly prevalent illness, both in the United States and worldwide, with a tremendous psychiatric, medical, and economic burden [1].Recently, there has been increased appreciation of the dynamic nature of depression, with several studies suggesting the clinical importance of temporal fluctuations of symptoms, including suicidal ideation [2,3]. However, the majority of clinical assessment tools for depressive symptoms are administered at fixed time points and subject to retrospective recall. Customized smartphone apps are a novel, low-cost means of conducting ecological momentary assessment to capture the temporal dynamics of depression.

The Patient Health Questionnaire-9 (PHQ-9) is a validated depression rating scale frequently utilized both in primary care [4] and psychiatry clinics. It assesses symptoms of depression over the preceding 2 weeks and is used for screening, diagnosing, and monitoring [5]. Numerous studies have demonstrated its utility in influencing clinical decision making [6,7], and meta-analyses have concluded that it has good diagnostic properties compared to longer screening tools [8]. However, like many scales, it is subject to patient retrospective recall, which may be especially biased in psychiatric illnesses [9].

Ecological momentary assessment (EMA) strives to complement static rating scales by offering *real-time* and *real-world* measurements of symptoms. In the past, this has often been conducted by asking patients to maintain paper-and-pencil mood logs. However, the labor-intensive and intrusive nature of paper-and-pencil EMA has limited its clinical applicability [10] and underscores the concern that data can be backfilled by patients [11]. Smartphones offer a potential solution by enabling EMA through apps designed to prompt, collect, time-stamp, and securely transfer patient data. EMA studies have been, and often still are, conducted on other devices such as Palm Pilots. Moving forward, we believe that conducting studies on smartphones is important because smartphone use may reduce bias in the form of the Hawthorne effect. This may occur if patients use their own devices, if devices are increasingly owned and used by mental health patients [12], and if the devices are both clinically applicable and scalable with today’s technology.

Although recent studies have demonstrated interest and ability of psychiatric patients to use smartphone apps for monitoring their mental health, there is currently no data on patients’ use of personal smartphones for depression EMA. In the general population, nearly 58% of US adults owned a smartphone in January 2014 [13] and this rate is expected to continue to increase. Recent evidence suggests that in patients with severe and persistent mental illness, personal smartphone ownership may be substantial [14]. In outpatient clinics, the percentage of patients with smartphones may even exceed the national average [15]. Feasibility and interest in utilizing smartphone apps to monitor mental illness has already been demonstrated in nearly all major diagnostic categories of psychiatric illness [16]. The proliferation of nearly 40,000 health apps in the iTunes and Android app stores suggests a strong interest among patients [17].

Patient adherence and patterns of daily use of smartphone apps by psychiatric patients is largely unknown. Efforts to assess rates of survey adherence include one depression study reporting that 68% of patients completed 75% of surveys [18]. A recent review of psychiatry-related mobile apps reported generally high adherence rates, notably higher than similar Internet-based interventions [16]. There is even less data on the daily patterns of use for these apps. One study of adolescents using a cognitive behavioral therapy (CBT) app to treat anxiety noted that the number of completed entries initially decreased during the first 3 weeks but then appeared to stabilize and remain fairly constant after week 4 [19].

Despite the ability of smartphone apps to now capture data in real time, there is still little known about how the symptoms of depression may vary in real time. Of particular interest is the potential of smartphone apps to be able to detect suicidal thoughts and offer new tools to better assess and understand suicidality.

While there is a burgeoning literature on smartphone apps and psychiatric illnesses, nearly all of these studies involve apps that are not available or accessible to all patients using the Android platform Google Play and Apple platform app stores. This may introduce unintended selection bias into studies. As noted in a recent study, patients refused to participate if the app was not able to run on their phone [20]. Although many studies of smartphone apps provide subjects with smartphones for the duration of the study, there is early evidence to suggest that patients may use their personal phone in a different manner than a loaned study phone [21].

We developed an iOS and Android smartphone app that assessed, three times a day, a randomized subset of three questions from the PHQ-9 questionnaire, including suicidality (see Methods for details). We chose to have the questions sampled from the PHQ-9 due to its broad applicability and demonstrated validity across numerous technologies, including touch screen computers [22], telephones [23], interactive voice technologies [24], and smartphones [25].

We hypothesized that psychiatric outpatients would be able to download the app to their personal smartphone and use it for 30 days. We further hypothesized that the smartphone-based EMA implementation would increase ease and convenience of data collection, as well as enable us to capture day-to-day variability in depressive symptoms and accurately represent intraindividual symptoms and their variation.

## Methods

### Design of Study and Smartphone App

Subjects were recruited from the outpatient psychiatry clinics at Beth Israel Deaconess Medical Center and the Massachusetts Mental Health Center, both located in Boston, Massachusetts. Inclusion criteria were a current diagnosis of major depressive disorder, current involvement in psychiatric treatment, and ownership of an iPhone or Android smartphone with the capacity to download and run the study app (ie, the subject was able to log in to the relevant app store, install the app on their own phone, and run the app). Further inclusion criteria were the agreement of the patient’s current mental health care provider that participation would not be harmful, and that they would review data collected from the app with the patient at the conclusion of the study. Exclusion criteria included patient refusal to participate or the psychiatric clinician’s concern that the subject was not appropriate for this study. The study duration was 29 or 30 days, depending on patient availability for a follow-up visit. On each day the subjects were asked to complete three short surveys delivered by the customized smartphone app. Subjects were notified that their responses on the app would not be monitored and would only be shared and reviewed when they met with their mental health providers after 29 or 30 days. In addition, each subject was asked to take the standard, paper-based PHQ-9 survey both at the beginning and at the end of the study period. The study protocol was approved by the Institutional Review Board (IRB) of Beth Israel Deaconess Medical Center and the Massachusetts Department of Mental Health. All participants signed an informed consent form.

**Figure 1:**
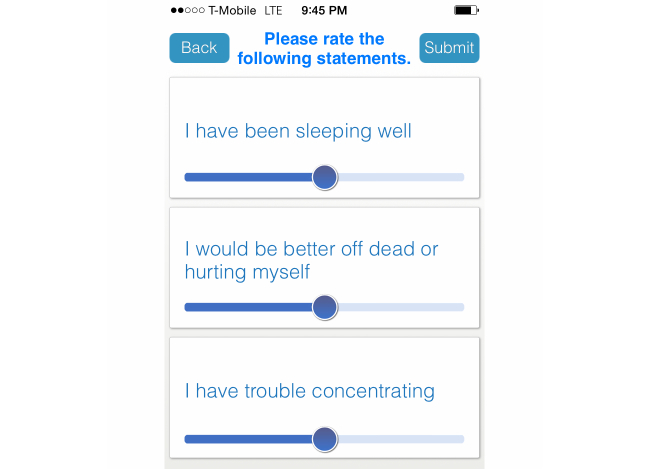
Screenshot of the Mindful Moods app. The app consisted only of a log-in screen and this survey screen on which randomized PHQ-9 questions were assessed at each survey session, three times a day.

Subjects were directed to download the app, Mindful Moods, onto their personal smartphone from either the Apple iTunes or Android Google Play store. The app alerted subjects that a survey was available three times per day at the following times: mornings between 10 AM and noon, afternoons between 1 PM and 3 PM, and evenings between 5 PM and 7 PM. Once alerted, subjects had a 2-hour time window to respond to the survey, after which time the survey was closed. In each survey session, subjects were asked to respond to three questions from the PHQ-9 questionnaire sampled without replacement and using a Likert scale (see Figure 1). Each survey session drew an independent sample of three questions from the PHQ-9 questionnaire, such that some questions were likely asked more than once and others not at all on a given day. Subjects were asked to reply to each question based on their experiences during the last day or 24-hour period. In order to increase patient engagement, assess response validity, and avoid survey fatigue, questions incorporated slight variations in wording, for example, if the subjects *were* feeling depressed or *were not* feeling depressed during different instances of the survey.

Subjects using the app had regular contact with their psychiatric clinicians, although neither they nor their clinicians had access to the patient mood data until the conclusion of the study. Although subjects were provided with contact information regarding questions about the app, no subject took advantage of such information. Subjects were compensated US $50 for completing at least 70% of survey sessions offered by the app.

### Statistical Estimation of Daily Patient Health Questionnaire-9 Scores

The PHQ-9 is usually administered on paper in a clinical setting, where patients are required to answer every question item. In this design, patients using the app might miss some questions over time. The app itself also incorporates randomness in the questions presented to increase response rates and potentially allow the estimation of additional survey features, such as within-day correlations between questions. These considerations invite novel estimation procedures for PHQ-9 scores.

A simple estimate of a patient’s PHQ-9 score is the sum of their daily responses, possibly weighted to adjust for data missingness. This naïve approach may be problematic as patients respond differently to various PHQ-9 questions, which are asked and/or answered at different rates, introducing bias. We, therefore, proposed a simple estimation strategy that incorporated question sampling and missingness by using information from the subject’s recent responses. The development of a more sophisticated statistical approach is part of our ongoing work.

We began by predicting the response of each question per day, which we estimated as the average response to that question over the last 2 weeks. Thus, before using any information from a given day itself, we obtained a prediction for the PHQ-9 score of a subject as the sum of predicted question responses. We then estimated a patient’s daily PHQ-9 score as this prediction *plus* the sum of differences between the predictions and the actual responses obtained throughout the day. This estimate had the desirable property of recovering the standard PHQ-9 score in the case that each PHQ-9 item happened to be asked and answered in a given day. Deviations from this standard yielded an estimate that borrowed information from past responses.

This method also admitted a natural estimate for the daily variance in PHQ-9 scores. For a given day, the variation in each question was estimated as the empirical variation in that question’s responses over the last 2 weeks. The variation in a daily PHQ-9 score was thereby estimated as the sum of the variances of the responses obtained for that day. From this estimate we were able to find confidence intervals for the predicted PHQ-9 score, yielding a statistical method for detecting if a daily score was significantly higher or lower than that predicted from previous variation. A more detailed description is given in Multimedia Appendix 1.

## Results

Out of a total of 14 patients who were offered the opportunity to participate, 13 (93%) enrolled in the study. The 13 subjects consisted of 3 males (23%) and 10 females (77%), with mean ages of 48 (SD 16) and 35 (SD 13), respectively. These 13 subjects took the initial in-office, paper-and-pencil PHQ-9 before downloading the app and their average PHQ-9 score was 8.62 (SD 4.56). We did not collect any data on the cognitive status and treatment course of any individual patient—all patients were currently diagnosed with, and in treatment for, major depressive disorder as confirmed by their mental health care providers.

**Figure 2:**
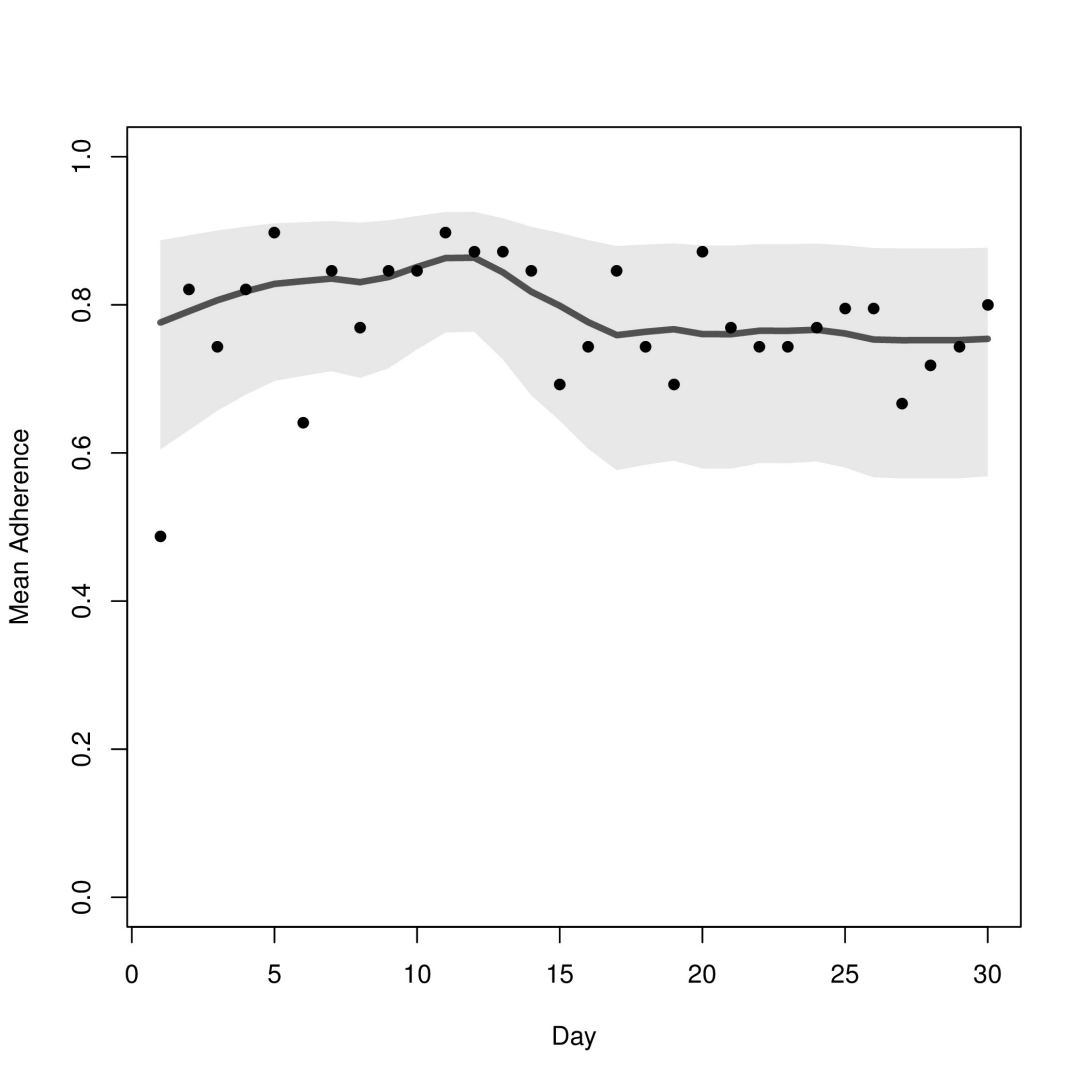
Average adherence to surveys delivered by the customized smartphone app over time (days). Responses were pooled from the three daily surveys: morning, afternoon, and evening. The gray band represents a 95% prediction interval.

We began with an assessment of the patient cohort adherence to the study protocol. Of the 13 subjects, 10 (77%) used the app for 30 days and 3 (23%) used the app for 29 days. Subjects were expected to respond to three short surveys per day, resulting in a total of 90 surveys per subject. A total of 903 of the 1161 short surveys administered to the cohort were completed, yielding an overall adherence of 77.78%. Adherence differed slightly with time of survey administration. The percentages of surveys completed were 75.5% (292/387) in the morning, 84.2% (326/387) in the afternoon, and 75.5% (292/387) in the evening. Analysis of variance showed a statistically significant difference in these adherence rates. Adherence remained strong throughout the study. As shown in Figure 2, adherence appeared to increase after a few days, decrease, and ultimately stabilize after about 2 weeks.

**Figure 3:**
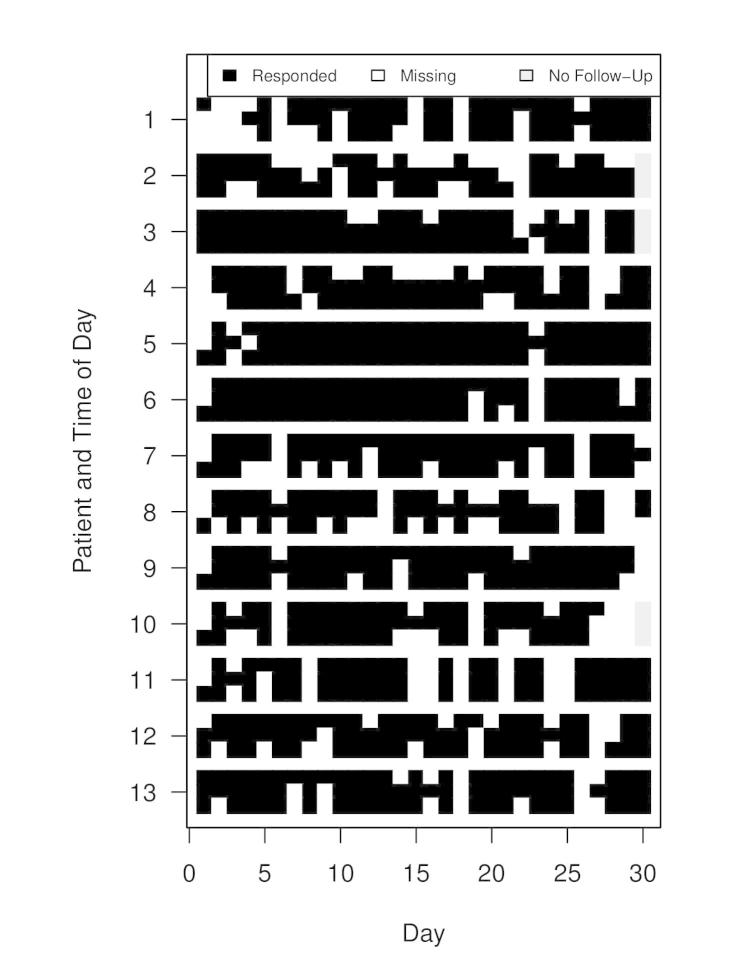
Adherence by patient, survey time, and day. Within patient rows, survey times are presented in columns of three, ordered as morning, afternoon, and evening from top to bottom, respectively.

A more detailed summary of adherence by patient, survey time, and day is shown in Figure 3. Although there is some variability in adherence across subjects, all subjects display surprisingly high adherence to the study protocol. The figure also suggests that there might be some subject-specific response patterns. For example, the pattern of missing surveys seems to vary from person to person—Subject 4 appears to be more likely to miss morning surveys than evening surveys, whereas Subject 8 is the opposite, appearing more likely to miss evening than morning surveys. In the figure, gray cells represent lack of data for patients who completed the study in 29 days—adherence was not calculated for these time points.

**Figure 4:**
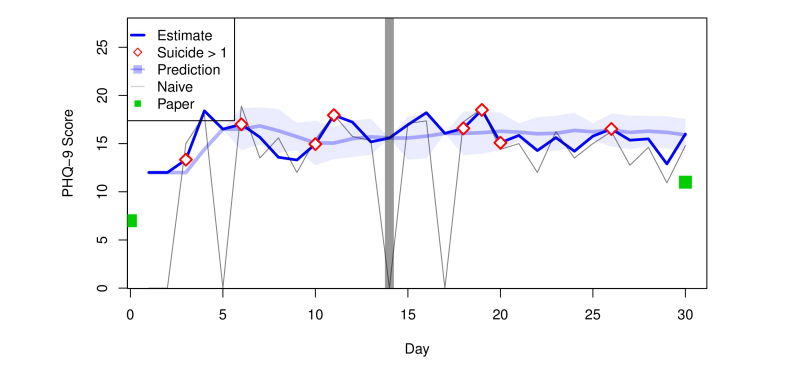
PHQ-9 estimates, reported suicidal thoughts, predictions, confidence intervals, naïve scores, and paper scores for Subject 1. Significant suicidal thoughts (Likert score >1) are shown as red diamonds. The green squares correspond to paper-based PHQ-9 scores. A horizontal line is included after the first 2 weeks.

As detailed in the Methods, the smartphone app presented the subjects with a sample of three PHQ-9 questions three times per day, where each sample was drawn with replacement from the nine questions of the PHQ-9 questionnaire. This approach was chosen to minimize subject fatigue by introducing variation in the questions asked. As a consequence of the study design, it was unlikely that each of the PHQ-9 questions would actually be asked on any given day, making it hard to reconstruct the PHQ-9 score from observed daily data alone. We reconstructed the PHQ-9 score using the statistical estimation approach described in the Methods. Figure 4 displays the daily PHQ-9 score prediction, confidence interval, and proposed estimate for Subject 1. Also indicated in the plot days is when the subject reported significant suicidal ideation, defined here as scoring 2 or 3 on Question 9 on suicidality.

**Figure 5:**
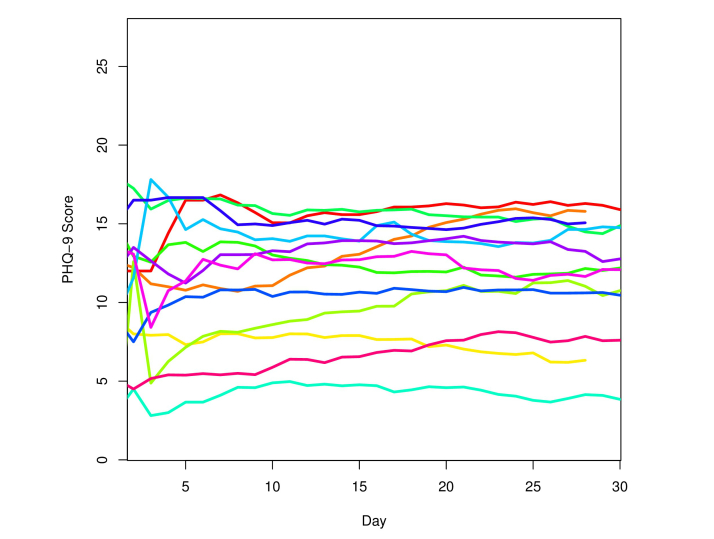
A composite plot of daily PHQ-9 score predictions. As weighted averages of recent scores, these predictions are much smoother than the estimates themselves.

A composite of information across patients is displayed in Figure 5, summarizing the PHQ-9 scores over time for each subject by plotting their score predictions. This plot illustrates the dynamic nature of PHQ-9 depression symptoms that can be captured with an app run on a subject’s personal smartphone.

**Figure 6:**
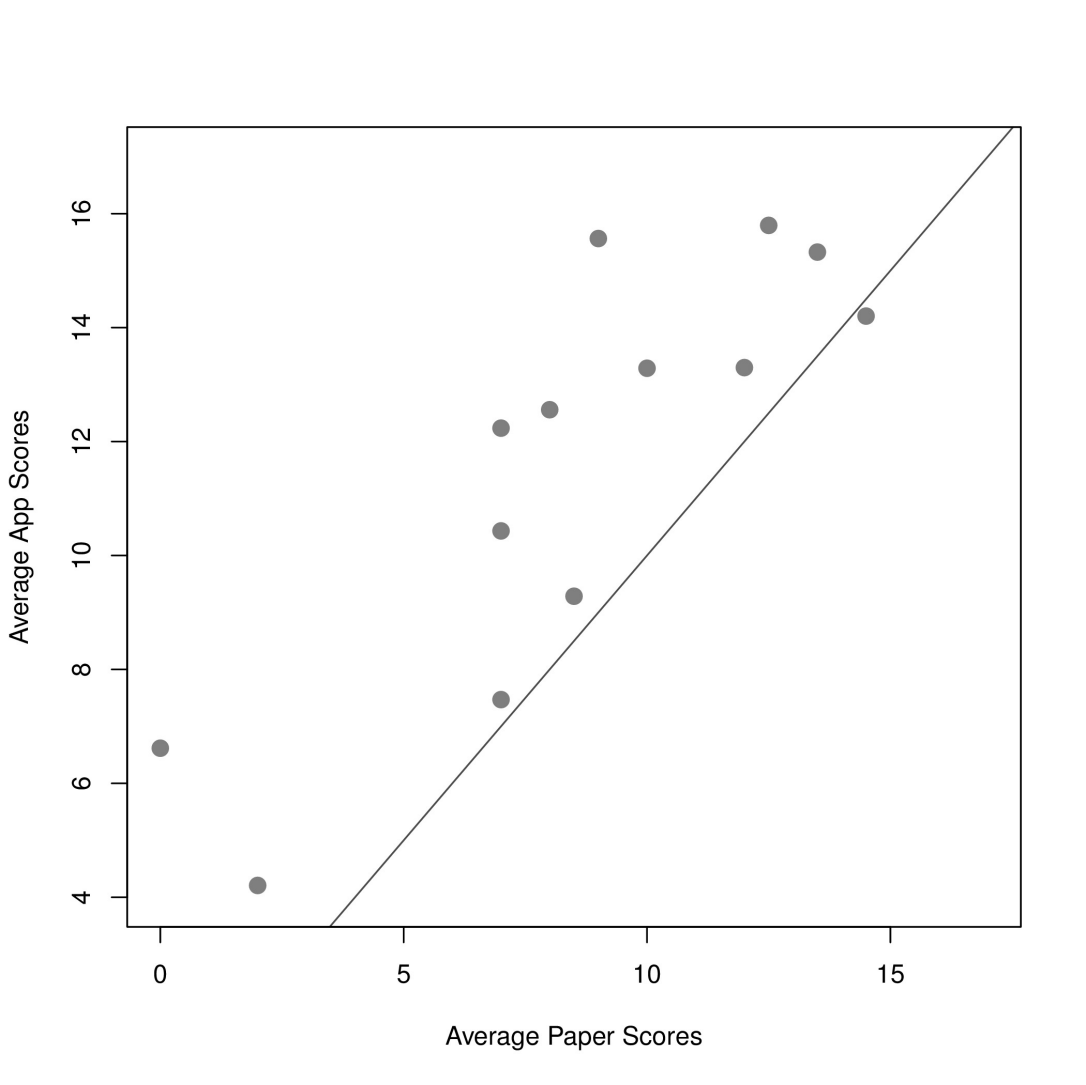
A plot of average paper and app PHQ-9 scores by patient (Pearson correlation coefficient, *r*=.84). Identical scores would lie on the added line.

Each patient also took a standard, paper-based PHQ-9. Those scores, taken at the beginning and end of the study, are also shown in Figure 4 (green squares). Returning to Subject 1, we see that the paper scores were lower than the app scores. This is generally true of patients in our study. Average paper and app scores for each subject are presented in Figure 6.

We found that the app scores were on average 3.02 (SD 2.25) points higher than paper scores. The two scores were fairly strongly correlated, yielding a Pearson correlation coefficient of .84 (95% CI .55-.95).

## Discussion

### Principal Findings

Our results suggest that it is feasible to monitor depressive symptoms in an outpatient setting utilizing a smartphone app running on a patient’s own phone. While prior studies have demonstrated similar results comparing paper and app PHQ-9 scores within a 2-hour time frame [26], our study employed three daily survey sessions in a 30-day period for a total of 90 survey sessions per subject. Other studies using apps to assess depressive symptoms have employed rating scales that included either single-point measurements with no follow-up [27,28], combined assessment with personalized feedback [25], or enhanced treatment [18,29]. These study designs have rendered it more difficult to understand the nature of depressive symptoms over an extended period of time independent of the study intervention.

While both the paper and app scores were strongly related, average PHQ-9 scores reported from the app were consistently higher. These results suggest that subjects with major depressive disorder may report a higher severity of symptoms with smartphones than to clinical providers. Potential explanations for this include the impact of retrospective recollection, patient desire to avoid embarrassment or disappointing their providers, or fear that reporting a higher burden of symptoms may lead to unwanted treatment changes or forced hospitalization. While our present data cannot address these hypotheses, we note that while no patient reported suicidality at a level of 2 or 3 on the paper-based PHQ-9, a total of 9 of the 13 subjects (69%) reported suicidal ideation on the app at these levels. The ability of the app to capture the presence of suicidal thoughts is an important result, suggesting that electronic monitoring may offer providers a more accurate method to record and understand suicidal ideation, as well as provide a means to prompt patients to call 911 if they report elevated suicidal scores. This ability of smartphones and related technologies to offer the mental health field a new tool in the detection, and in the potential prevention, of suicide is an important area for future study and development.

Our results provide novel information regarding psychiatric patients’ adherence with smartphone apps. The stability of the adherence rates over time suggests that patients are able to use smartphone apps for symptom collection over extended periods of time of at least 1 month. This corroborates the results of another study, in which adherence rates stabilized for the use a smartphone app to assess anxiety disorder [19]. Our data affords the opportunity to study adherence throughout the day. Adherence was shown to be higher in the afternoon compared to mornings and evenings, which indicates that afternoons might be the most opportune time to gather these kinds of data.

We did not provide novel monitoring devices or phones to patients, but rather utilized patients’ own smartphones. To the best of our knowledge, no prior study has enabled patients to use their personal smartphones, either Apple or Android models, to monitor their psychiatric symptoms. We believe that the high level of survey completion and lack of patient difficulty with the app may, in part, reflect the ease of app use on their personal phone instead of a study device. This suggests that future research efforts could enable patients to utilize their own smartphones, with substantial cost-saving for future research and clinical implementation.

Our results are also important as they underscore the utility of using only within-subject data to provide symptom scores, in this case the PHQ-9 estimates, which negates the need for large numbers of subjects in order to achieve statistical efficiency. As smartphones continue to develop with the addition of more sophisticated sensors, understanding the clinical uses and validating the clinical correlations of such data will likely become increasingly important.

### Limitations

There are several limitations to our study. While the sample size of 13 is small, it is comparable to similar and earlier pilot studies on this topic [25]. Patients in this study were not excluded due to comorbidity of additional illnesses. Our adherence rates are similar to other studies on mobile apps for patients with mental illness, however, it must be noted that subjects in this study might have been motivated, at least in part, by the US $50 that was contingent upon completing at least 70% of survey sessions. We also did not collect information on what type of treatment study subjects were receiving. In addition, our study lacked a control group, and it is possible that subject underreporting on the paper-and-pencil PHQ-9 might also have been a function of interacting with a clinician. Another potential confounder is the fact that subjects understood that their responses to the app would not be monitored, while reporting symptoms in the clinic on the paper scale received immediate mental health clinician review and attention.

### Conclusions

Our study demonstrates that subjects with a major depressive disorder are able to use a PHQ-9-based self-monitoring app on their personal smartphone, and that these results are correlated with traditional (paper-based) PHQ-9 scores. In addition, data collected through an app may potentially be both more sensitive to the symptoms of a major depressive disorder and better able to detect suicidal ideation. Finally, adherence with app use appears to be high, both throughout the day and over a 4-week time frame. These advantages suggest that digital monitoring of symptoms is feasible and provides an engaging, real-time, and low-cost supplement to the maintenance of mental health.

## Appendix

Multimedia Appendix 1 Additional methods.

## Abbreviations

CBT: cognitive behavioral therapy
EMA: ecological momentary assessment
IRB: Institutional Review Board
PHQ-9: Patient Health Questionnaire-9

## References

[1] Whiteford HA, Degenhardt L, Rehm J, Baxter AJ, Ferrari AJ, Erskine HE, Charlson FJ, Norman RE, Flaxman AD, Johns N, Burstein R, Murray CJ, Vos T (2013). Global burden of disease attributable to mental and substance use disorders: findings from the Global Burden of Disease Study 2010. Lancet.

[2] Selby EA, Yen S, Spirito A (2013). Time varying prediction of thoughts of death and suicidal ideation in adolescents: weekly ratings over 6-month follow-up. J Clin Child Adolesc Psychol.

[3] Handley TE, Attia JR, Inder KJ, Kay-Lambkin FJ, Barker D, Lewin TJ, Kelly BJ (2013). Longitudinal course and predictors of suicidal ideation in a rural community sample. Aust N Z J Psychiatry.

[4] Kroenke K, Spitzer RL, Williams JB (2001). The PHQ-9: validity of a brief depression severity measure. J Gen Intern Med.

[5] Löwe B, Kroenke K, Herzog W, Gräfe K (2004). Measuring depression outcome with a brief self-report instrument: sensitivity to change of the Patient Health Questionnaire (PHQ-9). J Affect Disord.

[6] Duffy FF, Chung H, Trivedi M, Rae DS, Regier DA, Katzelnick DJ (2008). Systematic use of patient-rated depression severity monitoring: is it helpful and feasible in clinical psychiatry?. Psychiatr Serv.

[7] Elliott T, Renier C, Palcher J (2013). PS1-29: PHQ-9 use in clinical practice: Electronic health record data at Essentia Health. Clin Med Res.

[8] Gilbody S, Richards D, Brealey S, Hewitt C (2007). Screening for depression in medical settings with the Patient Health Questionnaire (PHQ): a diagnostic meta-analysis. J Gen Intern Med.

[9] Takayanagi Y, Spira AP, Roth KB, Gallo JJ, Eaton WW, Mojtabai R (2014). Accuracy of reports of lifetime mental and physical disorders: results from the Baltimore Epidemiological Catchment Area study. JAMA Psychiatry.

[10] aan het Rot M, Hogenelst K, Schoevers RA (2012). Mood disorders in everyday life: a systematic review of experience sampling and ecological momentary assessment studies. Clin Psychol Rev.

[11] Depp CA, Kim DH, de Dios LV, Wang V, Ceglowski J (2012). A pilot study of mood ratings captured by mobile phone versus paper-and-pencil mood charts in bipolar disorder. J Dual Diagn.

[12] Torous J, Chan SR, Yee-Marie Tan S, Behrens J, Mathew I, Conrad EJ, Hinton L, Yellowlees P, Keshavan M (2014). Patient smartphone ownership and interest in mobile apps to monitor symptoms of mental health conditions: A survey in four geographically distinct psychiatric clinics. JMIR Mental Health.

[13] Pew Research Center.

[14] Ben-Zeev D, Brenner CJ, Begale M, Duffecy J, Mohr DC, Mueser KT (2014). Feasibility, acceptability, and preliminary efficacy of a smartphone intervention for schizophrenia. Schizophr Bull.

[15] Torous J, Friedman R, Keshavan M (2014). Smartphone ownership and interest in mobile applications to monitor symptoms of mental health conditions. JMIR Mhealth Uhealth.

[16] Donker T, Petrie K, Proudfoot J, Clarke J, Birch M-R, Christensen H (2013). Smartphones for smarter delivery of mental health programs: a systematic review. J Med Internet Res.

[17] Powell AC, Landman AB, Bates DW (2014). In search of a few good apps. JAMA.

[18] Schaffer A, Kreindler D, Reis C, Levitt AJ (2013). Use of mental health telemetry to enhance identification and predictive value of early changes during augmentation treatment of major depression. J Clin Psychopharmacol.

[19] Pramana G, Parmanto B, Kendall PC, Silk JS (2014). The SmartCAT: an m-health platform for ecological momentary intervention in child anxiety treatment. Telemed J E Health.

[20] Faurholt-Jepsen M, Frost M, Vinberg M, Christensen EM, Bardram JE, Kessing LV (2014). Smartphone data as objective measures of bipolar disorder symptoms. Psychiatry Res.

[21] Ainsworth J, Palmier-Claus JE, Machin M, Barrowclough C, Dunn G, Rogers A, Buchan I, Barkus E, Kapur S, Wykes T, Hopkins RS, Lewis S (2013). A comparison of two delivery modalities of a mobile phone-based assessment for serious mental illness: native smartphone application vs text-messaging only implementations. J Med Internet Res.

[22] Fann JR, Berry DL, Wolpin S, Austin-Seymour M, Bush N, Halpenny B, Lober WB, McCorkle R (2009). Depression screening using the Patient Health Questionnaire-9 administered on a touch screen computer. Psychooncology.

[23] Pinto-Meza A, Serrano-Blanco A, Peñarrubia MT, Blanco E, Haro JM (2005). Assessing depression in primary care with the PHQ-9: can it be carried out over the telephone?. J Gen Intern Med.

[24] Turvey C, Sheeran T, Dindo L, Wakefield B, Klein D (2012). Validity of the Patient Health Questionnaire, PHQ-9, administered through interactive-voice-response technology. J Telemed Telecare.

[25] Burns MN, Begale M, Duffecy J, Gergle D, Karr CJ, Giangrande E, Mohr DC (2011). Harnessing context sensing to develop a mobile intervention for depression. J Med Internet Res.

[26] Bush NE, Skopp N, Smolenski D, Crumpton R, Fairall J (2013). Behavioral screening measures delivered with a smartphone app: psychometric properties and user preference. J Nerv Ment Dis.

[27] Pelletier JF, Rowe M, François N, Bordeleau J, Lupien S (2013). No personalization without participation: on the active contribution of psychiatric patients to the development of a mobile application for mental health. BMC Med Inform Decis Mak.

[28] Webb JR, Webb BF, Schroeder MC, North CS (2013). Association of aphthous ulcers with self-reported symptoms of depression in a sample of smartphone users. Ann Clin Psychiatry.

[29] Ly KH, Trüschel A, Jarl L, Magnusson S, Windahl T, Johansson R, Carlbring P, Andersson G (2014). Behavioural activation versus mindfulness-based guided self-help treatment administered through a smartphone application: a randomised controlled trial. BMJ Open.

