# Supplementary material for: Utilizing a Personal Smartphone Custom App to Assess the Patient Health Questionnaire-9 (PHQ-9) Depressive Symptoms in Patients With Major Depressive Disorder
Source: JMIR Ment Health. 2015 Mar 24;2(1):e8. doi: 10.2196/mental.3889 (PMC4607379; doi:10.2196/mental.3889)
Supplement: Multimedia Appendix 1 [file mental_v2i1e8_app1.pdf]

Online Supplementary Material

This supplement contains detailed information regarding the quantitative analysis for our study. We present and compare the questions asked by the Mindful Moods application as well as questions posed in the standard, paper-based PHQ-9 test. We also detail the statistical methods used to estimate daily PHQ-9 scores as well as their associated variance. Finally, we summarize this information for a sample patient.

Survey Questions

In this section, we will detail the questions and assumptions used in our study.

The PHQ-9 asks nine questions with the intent of assessing the severity of depression. These questions are answered on a 0-3 Likert scale, with increasing scores indicating increased severity. Questions presented in the PHQ-9 are phrased retrospectively, asking patients to assess the severity of their symptoms over the last two weeks. The difference between these perspectives is included in the estimation procedures that follow.

The Mindful Moods application adapts the PHQ-9 for Ecological Momentary Assessment (EMA). For each of the nine retrospective questions asked in the PHQ-9, Mindful Moods asks analogous questions that are phrased to assess the severity of symptoms in the present or recent past. Furthermore, in order to make Mindful Moods more engaging, each question has two phrasings with opposite valence, and one phrasing is selected at random for each question asked. These questions are assumed to be interchangeable after tranforming scores with opposite valence to match that of the original PHQ-9. Specifically, if a subject responds with a score of Y to a Mindful Moods question with the same valence as its analogous PHQ-9 question, the oppositely phrased version is assumed to be comparable if transformed as $3 - Y$.

Table 1 provides both the original phrasing of the PHQ-9 (as well as the retrospective preamble) and the two versions of each question posed the Mindful Moods application.

	**Original PHQ-9**: *Over the past 2 weeks, how often have you been bothered by any of the following problems?*	**Mindful Moods**<br>• Version 1 (no preamble)<br>• Version 2 (no preamble)
Question 1	• Little interest or pleasure in doings things	• I have little interest or please in doing things<br>• I have lots of interest or please in doing things
Question 2	• Feeling down, depressed, or hopeless	• I feel down and depressed and hopeless<br>• I feel up and bright and hopeful
Question 3	• Trouble falling or staying asleep, or sleeping too much	• I have trouble with sleep<br>• I have been sleeping well
Question 4	• Feeling tired or having little energy	• I have been feeling tired and have little energy<br>• I have been feeling active and have lots of energy
Question 5	• Poor appetite or overeating	• I have a poor appetite or am overeating<br>• I am eating the right amount of food
Question 6	• Feeling bad about yourself—or that you are a failure or have let yourself or your family down	• I feel guilty or bad about myself<br>• I feel positive and good about myself
Question 7	• Trouble concentrating on thins, such as reading the newspaper or watching television	• I have trouble concentrating<br>• I can concentrate well
Question 8	• Moving or speaking so slowly that other people could have noticed. Or the opposite—being so fidgety or restless that you have been moving around a lot more than usual	• I am moving slower or fidgeting more<br>• I am not fidgety or feel weighed down either
Question 9	• Thoughts that you would be better off dead, or of hurting yourself	• I would be better off dead or hurting myself<br>• I do not want to hurt or kill myself

Table 1: Questions from the original PHQ-9 survey as well as both versions of questions from Mindful Moods.

Estimation

The PHQ-9 asks each of its nine questions at one time, and the administrator requires the subject to give a response to each question. In contrast, the Mindful Moods application is a continual momentary ecological assessment, and patients might not adhere to all nine questions if asked repeatedly over time. To this end, the application asks only a subset of its questions throughout the day, with the goal of achieving high response rates over time. However, because only a subset of the questions are asked at a given time, the complete momentary PHQ-9 score requires estimation.

Let these responses to application questions be notated $Y_{tq} = 0, ..., 3$, where $q = 1, ..., 9$ indexes the question and $t = 1, ..., T$ represents the day the question was asked. Thus, if every question was asked and answered, a daily PHQ-9 score is exactly $P_t = \sum_q Y_{tq}$. In our scheme, 3 sets of questions were asked throughout the day, and each set independently sampled 3 questions without replacement from $1, ..., 9$, and the app therefore measures $Y_{ta_{tq}}$, where each $a_{tq} \in \{1, ..., 9\}$. Thus, on most days not every question will be asked, and some will be asked more than once. We wish to estimate P_t as $\widehat{P}_t$ with this information.

If our estimate of a patient's daily PHQ-9 score was a naive sum of the questions we asked, the estimate will be biased because a patient might reliably score higher on some questions than others, so this estimate of PHQ-9 could reflect either how they feel or what we asked. Furthermore, we must account for missing data, which might be common with EMA questionaires. Another estimate is required that incorporates this heterogeneity of the data.

Our approach is to predict a subject's likely responses to each PHQ-9 question for that day, and to replace this prediction when actual responses are given for questions asked. Because PHQ-9 questions are phrased retrospectively and Mindful Moods questions are asked momentarily, we assume that the best prediction for a PHQ-9 question response $\widetilde{Y}_{tq}$ is the average of Mindful Moods responses given for the same question item over the last two weeks:

$$\widetilde{Y}_{tq} = \frac{\sum_{i=\max(1,t-14)}^{t} \sum_{k=1}^{9} \mathbb{I}(a_{ik} = q) Y_{ia_{ik}}}{\sum_{i=\max(1,t-14)}^{t} \sum_{k=1}^{9} \mathbb{I}(a_{ik} = q)} \tag{1}$$

This leads to an *a priori* prediction for each day's PHQ-9 score $\widetilde{P}_t = \sum_{q=1}^{9} \widetilde{Y}_{tq}$. We then estimate the patient's PHQ-9 score as this predicted score, plus the total difference between that prediction and the day's measured responses:

$$\widehat{P}_t = \sum_{q=1}^{9} \left(\widetilde{Y}_{tq} + \left[Y_{ta_{tq}} - \widetilde{Y}_{ta_{tq}} \right] \right) \tag{2}$$

In the special case that all 9 questions are asked and answered daily, $a_{tq} = q$, so $\widehat{P}_t = P_t$. This also acts as an imputation for questions or days missing at random.

Inference

The severity of depression symptoms varies over time, so a momentary PHQ-9 score is expected to vary over time as well. This variance can be used to assess how unusual a subject's daily PHQ-9 score is when compared to variation in previous scores. We can capture this variation in estimated daily PHQ-9 scores by using the variation in recent responses to Mindful Moods question items. We begin by first estimating the variance in question responses Y_{tq} as the maximum likelihood variance in responses over the last two weeks:

$$\widehat{\mathrm{Var}}(Y_{tq}) = \frac{\sum_{i=\max(1,t-14)}^{t} \sum_{k=1}^{9} \mathbb{I}(a_{ik} = q) \left[Y_{ia_{ik}} - \widetilde{Y}_{ia_{ik}} \right]^2}{\left[\sum_{i=\max(1,t-14)}^{t} \sum_{k=1}^{9} \mathbb{I}(a_{ik} = q) \right] - 1} \tag{3}$$

Assuming the responses to questions are independent and that the predicted responses $\widetilde{Y}_{tq}$ are approximately fixed, the variance in P_t may therefore be estimated as the sum of variance in the given responses:

$$\widehat{\mathrm{Var}}(\widehat{P}_t) = \widehat{\mathrm{Var}}(\sum_{q=1}^{9} Y_{ta_{tq}}) \approx \sum_{q=1}^{9} \widehat{\mathrm{Var}}(Y_{ta_{tq}}) \tag{4}$$

Assuming estimated PHQ-9 scores $\widehat{P}_t$ are approximately normal, we may proceed to construct level $100(1-\alpha)\%$ confidence intervals and level α tests for the daily PHQ-9 score:

$$\widehat{\mathrm{CI}}(\widehat{P}_t) = \widetilde{P}_t \pm Z_{1-\alpha/2} \sqrt{\widehat{\mathrm{Var}}(\widehat{P}_t)} \tag{5}$$

Thus $\widehat{P}_t$ falls outside this region with estimated minimum probability α. We traditionally consider this an error rate. However, in our context, α represents the approximate proportion of unusual daily PHQ-9 scores.

Results

Many of the considerations detailed in previous sections can be summarized in a single plot for each patient. For example, PHQ-9 predictions and estimates $\widetilde{P}_t$ and $\widehat{P}_t$ are defined for each day of the study, with the most information being used after the first two weeks of gathering data. Confidence intervals can also be constructed for each day. These results can be compared to naïve averages of daily question responses. A summary of this information is shown for Patient 1 in our dataset in Figure 1. The vertical gray bar indicates the two week marker, after which time the maximum information is used for estimates and confidence intervals, so daily results reflect the most information. The plot displays significant scores for questions assessing suicidal tendencies (when $Y_{t9} = 2$ or 3), shown with red diamonds. For comparison, the plot also shows complete paper PHQ-9 assessments in green, which were administered at days 1 and 30.

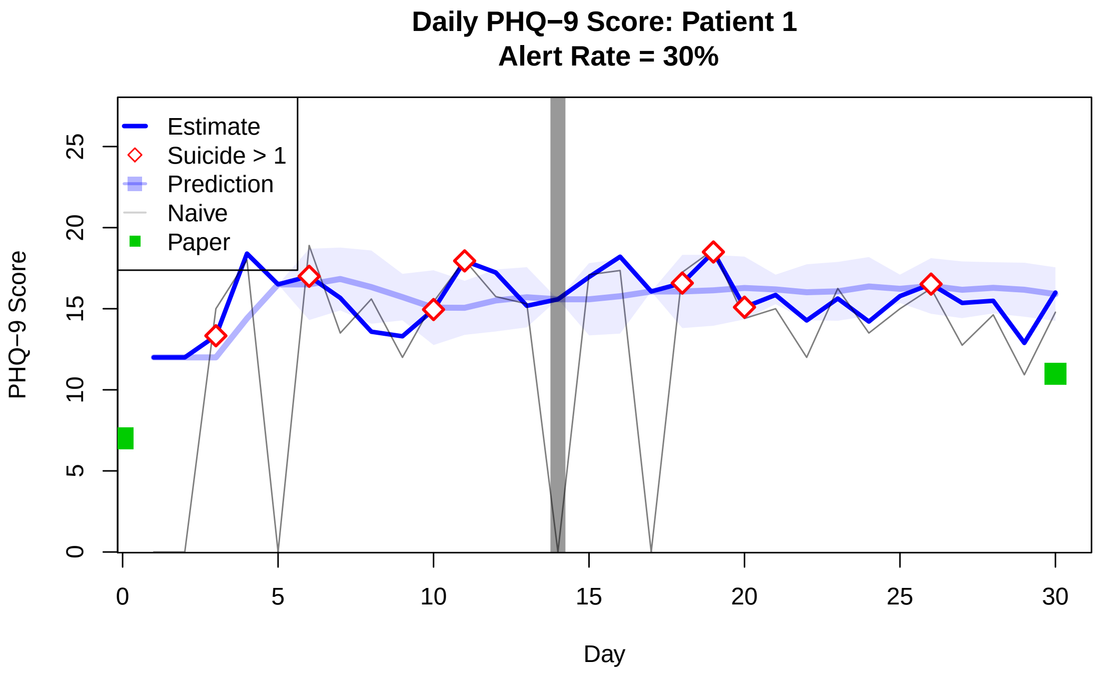

Figure 1: Daily PHQ-9 predictions, confidence intervals, and estimates for Patient 1. Naïve daily averages are included for comparison. Significant suicidal thoughts are shown in red, and paper PHQ-9 scores are shown in green.
